# Crystal structure of methyl 4-(4-hy­droxy­phen­yl)-6-methyl-2-oxo-1,2,3,4-tetra­hydro­pyrimidine-5-carboxyl­ate monohydrate

**DOI:** 10.1107/S2056989016013359

**Published:** 2016-08-26

**Authors:** Keshab M. Bairagi, Katharigatta N. Venugopala, Pradip Kumar Mondal, Bharti Odhav, Susanta K. Nayak

**Affiliations:** a Department of Chemistry, Visvesvaraya National Institute of Technology, Nagpur, 440010, Maharashtra, India; b Department of Biotechnology and Food Technology, Durban University of Technology, Durban 4001, South Africa; c Department of Chemistry, Indian Institute of Science Education and Research Bhopal, Bhauri, Bhopal 462023, India

**Keywords:** crystal structure, DHPM derivative, monohydrate, *Z*′ = 2, hydrogen bonding

## Abstract

The title hydrate, C_13_H_14_N_2_O_4_·H_2_O, crystallizes with two formula units in the asymmetric unit (*Z*′ = 2). The dihedral angles between the planes of the tetra­hydro­pyrimidine ring and the 4-hy­droxy­phenyl ring and ester group are 86.78 (4) and 6.81 (6)°, respectively, for one mol­ecule and 89.35 (4) and 3.02 (4)° for the other. In the crystal, the organic mol­ecules form a dimer, linked by a pair of N—H⋯O hydrogen bonds. The hydroxy groups of the organic mol­ecules donate O—H⋯O hydrogen bonds to water mol­ecules. Further, the hy­droxy group accepts N—H⋯O hydrogen bonds from amides whereas the water mol­ecules donate O—H⋯O hydrogen bonds to the both the amide and ester carbonyl groups. Other weak inter­actions, including C—H⋯O, C—H⋯π and π–π, further consolidate the packing, generating a three-dimensional network.

## Abstract 2

The title hydrate crystallizes with two formula units in the asymmetric unit (*Z*′ = 2). The organic mol­ecules form a dimer, linked by a pair of N—H⋯O hydrogen bonds. Further hydrogen bonding together with weak C—H⋯π and π–π inter­actions further consolidates the packing, generating a three-dimensional network.

## Chemical context   

Di­hydro­pyrimidine (DHPM) derivatives are used in the treatment of disease as anti­viral, anti­tumor, anti­bacterial and anti­malarial agents, as first reported by the Italian chemist Pietro Biginelli in 1893 [Kappe (2000 ▸), Nayak *et al.* (2010 ▸) and references therein]. We have been working on the synthesis of various DHPM derivatives for better biological activities (Narayanaswamy *et al.*, 2013 ▸; Nayak *et al.*, 2011 ▸) and a wide range of applications (Nayak *et al.*, 2009 ▸, 2010 ▸). Here, we report the synthesis and single-crystal structure of the title compound, (I).
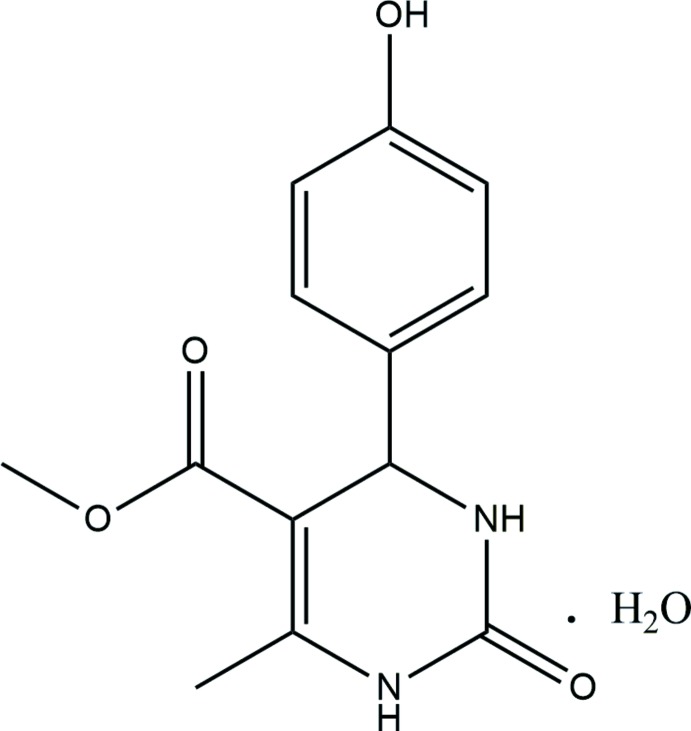


## Structural commentary   

**Figure 1:**
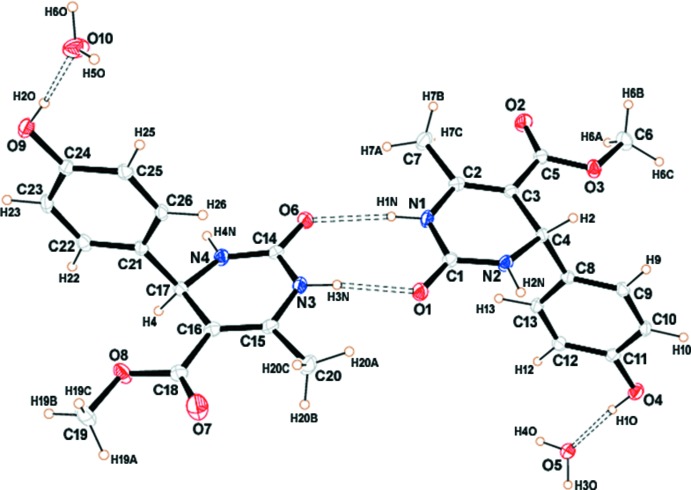
The asymmetric unit of the title compound with 50% probability ellipsoids. The double-dashed lines indicate hydrogen bonds.

Compound (I) crystallizes as a monohydrate with two formula units in the asymmetric unit (*Z*′ = 2), which may be supported by the formation of hydrogen bonds between the hy­droxy­phenyl group and the water mol­ecule and dimer formation through N—H⋯O hydrogen bonds (Fig. 1 ▸). The dihedral angles between the planes of the six-membered tetra­hydro­pyrimidine ring with its 4-hy­droxy­phenyl and ester substituents are 86.78 (4) and 6.81 (6)°, respectively, for the N1-containing mol­ecule and 89.35 (4)° and 3.02 (4)°, respectively, for the other.

## Supra­molecular features   

**Figure 2:**
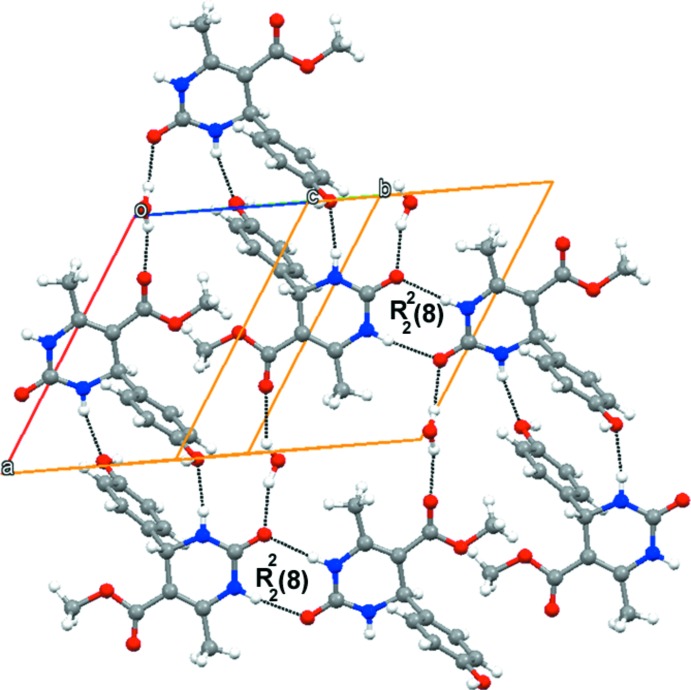
Crystal structure of title compound showing the dimers formed by N—H⋯O hydrogen bonds as well as the links to the water mol­ecules, which donate O—H⋯O hydrogen bonds to the ester groups.

**Figure 3:**
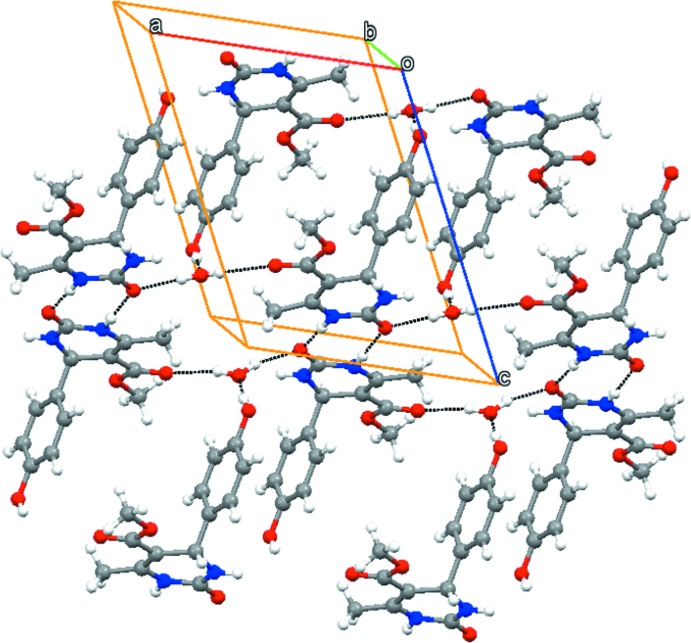
Three-dimensional crystal structure of the title compound showing the role of the water mol­ecules in the hydrogen-bonding network.

In the crystal of (I), the DHPM mol­ecules form dimers through N—H⋯O hydrogen bonds with an 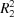
(8) graph-set motif (Fig. 2 ▸). The hy­droxy groups of the di­hydro­pyrimidine mol­ecules donate O—H⋯O hydrogen bonds to water mol­ecules, which may explain the preference for the monohydrated crystalline form. Further, the hy­droxy group accepts N—H⋯O hydrogen bonds from amide groups whereas the water mol­ecule donates O—H⋯O hydrogen bonds to the both the amide and ester carbonyl groups (Table 1 ▸). The key role of the water mol­ecule in the hydrogen-bonding network is shown in Fig. 3 ▸.

Weak inter­actions including C—H⋯O, C—H⋯π and π⋯π [*Cg*1⋯*Cg*2(2 − *x*, 1 − *y*, 1 − *z*) = 3.652 (1) Å; *Cg*1 and *Cg*2 are the centroids of the C8–C13 and C21–C26 rings, respectively] help to consolidate the packing and a three-dimensional network arises.

## Database survey   

A search of the Cambridge structural Database (CSD) (*Conquest* Version 1.17; Groom *et al.*, 2016 ▸) for methyl 4-(4-hy­droxy­phen­yl)-6-methyl 2-oxo-1,2,3,4-tetra­hydro­pyrimidine-5-carboxyl­ate gave no hits; however, the crystal structures of sixteen hy­droxy­phenyl-substituted DHPM derivatives were found. These structures include four 2-hy­droxy­phenyl-substituted DHPM mol­ecules, one 3-hy­droxy-substituted and eleven 4-hy­droxy­phenyl-substituted DHPM mol­ecules. It is inter­esting to note that five of the 4-hy­droxy­phenyl-substituted DHPM mol­ecules prefer to crystallize in a hydrated form (ZOHFIN: Vishnevskii *et al.* 2014 ▸; VOJDOO: Das *et al.*, 2008 ▸; VOJDOO01: Nayak *et al.*, 2009 ▸; POWXIJ: Thenmozhi *et al.*, 2009 ▸; XISMES: Liu *et al.*, 2008 ▸). However, of these only ethyl 4-(4-hy­droxy­phen­yl)-6-methyl-2-oxo-1,2,3,4-tetra­hydro­pyrimidine-5 carboxyl­ate (VOJDOO: Das *et al.*, 2008 ▸) crystallizes with a higher formula unit (*Z*′ > 1), *i.e.* its structure has three formula units in the asymmetric unit (*Z*′ = 3) in the monohydrated form. Hence, the title compound is the second member of this family of monohydrates to crystallize with higher formula units in the asymmetric unit (*Z*′ = 2). The CSD analysis clearly suggests that 4-hy­droxy-substituted DHPM mol­ecule are prone to crystallize in their hydrated form compared to 3-hy­droxy or 2-hy­droxy-substituted DHPM mol­ecules; this may be due to the observed O—H⋯O hydrogen bonding with water mol­ecule acceptors with the hydroxyl group in the preferred *para* position.

## Synthesis and crystallization   

The title compound was obtained by the reaction of three components, *viz*. methyl aceto­acetate, 4-hy­droxy­benzaldehyde and urea in ethanol solution according to a reported procedure (Tumtin *et al.*, 2010 ▸). The reaction progress was monitored by thin layer chromatography and after the completion of the reaction, the solvent was removed and the solid obtained was recrystallized from ethanol to obtain the pure product. Colorless single crystals suitable for X-ray diffraction analysis were obtained by slow evaporation of a solution in ethanol (yield 75%, m.p. 412.3 K). FT–IR ν_max_ cm^−1^: 3379 (O—H), 3248 (N—H), 2963 (*sp* ^2^ C—H), 2845 (*sp* ^3^ C—H), 1763 (C=O ester), 1682 (C=O amide), 1594 (C=C alkene), 1514 (C=C aromatic) and 1260 (C—O, ester).

## Refinement   

Crystal data, data collection and structure refinement details are summarized in Table 2 ▸. All hydrogen atoms were located in difference Fourier maps and freely refined.

## Supplementary Material

Crystal structure: contains datablock(s) I. DOI: 10.1107/S2056989016013359/hb7604sup1.cif

Structure factors: contains datablock(s) I. DOI: 10.1107/S2056989016013359/hb7604Isup2.hkl

Supporting information file. DOI: 10.1107/S2056989016013359/hb7604Isup3.cml

CCDC reference: 1499989

Additional supporting information: crystallographic information; 3D view; checkCIF report

## Figures and Tables

**Table 1:** Hydrogen-bond geometry (Å, °) *Cg*1 and *Cg*2 are the centroids of the C8–C13 and C21–C26 rings, respectively.

*D*—H⋯*A*	*D*—H	H⋯*A*	*D*⋯*A*	*D*—H⋯*A*
N1—H1*N*⋯O6	0.878 (19)	2.10 (2)	2.9762 (16)	173.1 (17)
N3—H3*N*⋯O1	0.88 (2)	1.98 (2)	2.8626 (16)	174 (2)
N2—H2*N*⋯O9^i^	0.89 (2)	2.02 (2)	2.8971 (19)	170.0 (18)
N4—H4*N*⋯O4^ii^	0.87 (2)	2.13 (2)	2.9738 (19)	163.4 (18)
O4—H1*O*⋯O5	0.87 (2)	1.78 (2)	2.6473 (16)	175 (2)
O9—H2*O*⋯O10	0.90 (2)	1.73 (2)	2.6189 (17)	174.7 (19)
O5—H4*O*⋯O2^iii^	0.84 (2)	2.15 (2)	2.8549 (19)	141 (2)
O5—H3*O*⋯O1^iv^	0.91 (3)	1.91 (3)	2.786 (2)	162 (2)
C20—H20*C*⋯O5^iv^	1.00 (2)	2.56 (2)	3.332 (2)	133.7 (17)
C26—H26⋯O3^v^	0.971 (17)	2.571 (18)	3.4607 (18)	152.4 (14)
C6—H6*B*⋯*Cg*2^vi^	0.989 (19)	2.70 (2)	3.392 (2)	127.1 (16)
C19—H19*C*⋯*Cg*1^vii^	0.98 (2)	2.84 (2)	3.395 (2)	116.5 (16)

Symmetry codes: (i) 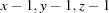; (ii) 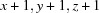; (iii) 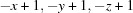; (iv) 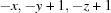; (v) 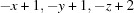; (vi) 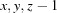; (vii) 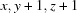.

**Table 2:** Experimental details

Crystal data	
Chemical formula	C_13_H_14_N_2_O_4_·H_2_O
*M*_r_	280.28
Crystal system, space group	Triclinic, *P* 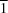
Temperature (K)	150
*a*, *b*, *c* (Å)	10.7527 (6), 11.6731 (6), 12.4456 (7)
α, β, γ (°)	98.236 (2), 112.374 (1), 108.944 (2)
*V* (Å^3^)	1301.16 (13)
*Z*	4
Radiation type	Mo *K*α
μ (mm^−1^)	0.11
Crystal size (mm)	0.23 × 0.20 × 0.15

Data collection	
Diffractometer	Bruker Kappa APEXII DUO
Absorption correction	Multi-scan (*SADABS*; Bruker, 2008 ▸)
*T*_min_, *T*_max_	0.926, 0.934
No. of measured, independent and observed [*I* > 2σ(*I*)] reflections	21716, 5116, 4387
*R*_int_	0.044
(sin θ/λ)_max_ (Å^−1^)	0.617

Refinement	
*R*[*F* ^2^ > 2σ(*F* ^2^)], *wR*(*F* ^2^), *S*	0.038, 0.099, 1.04
No. of reflections	5116
No. of parameters	489
H-atom treatment	All H-atom parameters refined
Δρ_max_, Δρ_min_ (e Å^−3^)	0.23, −0.28

Computer programs: *APEX2* and *SAINT* (Bruker, 2008 ▸), *SHELXS97* (Sheldrick, 2008 ▸), *SHELXL2014/7* (Sheldrick, 2015 ▸), *Mercury* (Farrugia, 2012 ▸) and *ORTEP-3 for Windows* (Macrae *et al*, 2008 ▸).

## supplementary crystallographic information

### Crystal data

**Table:**

C_13_H_14_N_2_O_4_·H_2_O	*Z* = 4
*M**_r_* = 280.28	*F*(000) = 592
Triclinic, *P*1	*D*_x_ = 1.431 Mg m^−^^3^
*a* = 10.7527 (6) Å	Mo *K*α radiation, λ = 0.71073 Å
*b* = 11.6731 (6) Å	Cell parameters from 740 reflections
*c* = 12.4456 (7) Å	θ = 2.2–30.1°
α = 98.236 (2)°	µ = 0.11 mm^−^^1^
β = 112.374 (1)°	*T* = 150 K
γ = 108.944 (2)°	Plate, colorless
*V* = 1301.16 (13) Å^3^	0.23 × 0.20 × 0.15 mm

### Data collection

**Table:**

Bruker Kappa APEXII DUO diffractometer	5116 independent reflections
Radiation source: fine-focus sealed tube	4387 reflections with *I* > 2σ(*I*)
Graphite monochromator	*R*_int_ = 0.044
ω scans	θ_max_ = 26.0°, θ_min_ = 2.3°
Absorption correction: multi-scan (SADABS; Bruker, 2008)	*h* = −13→13
*T*_min_ = 0.926, *T*_max_ = 0.934	*k* = −14→14
21716 measured reflections	*l* = −15→15

### Refinement

**Table:**

Refinement on *F*^2^	Primary atom site location: structure-invariant direct methods
Least-squares matrix: full	Secondary atom site location: difference Fourier map
*R*[*F*^2^ > 2σ(*F*^2^)] = 0.038	Hydrogen site location: difference Fourier map
*wR*(*F*^2^) = 0.099	All H-atom parameters refined
*S* = 1.04	*w* = 1/[σ^2^(*F*_o_^2^) + (0.046*P*)^2^ + 0.5474*P*] where *P* = (*F*_o_^2^ + 2*F*_c_^2^)/3
5116 reflections	(Δ/σ)_max_ = 0.001
489 parameters	Δρ_max_ = 0.23 e Å^−^^3^
0 restraints	Δρ_min_ = −0.28 e Å^−^^3^

### Special details

**Table:**

Geometry. All esds (except the esd in the dihedral angle between two l.s. planes) are estimated using the full covariance matrix. The cell esds are taken into account individually in the estimation of esds in distances, angles and torsion angles; correlations between esds in cell parameters are only used when they are defined by crystal symmetry. An approximate (isotropic) treatment of cell esds is used for estimating esds involving l.s. planes.

### Fractional atomic coordinates and isotropic or equivalent isotropic displacement parameters (Å^2^)

**Table:**

	*x*	*y*	*z*	*U*_iso_*/*U*_eq_	
C1	0.39164 (15)	0.54081 (12)	0.87596 (12)	0.0139 (3)	
C2	0.60979 (15)	0.53784 (13)	0.86203 (12)	0.0148 (3)	
C3	0.53110 (14)	0.42096 (12)	0.77764 (12)	0.0136 (3)	
C4	0.36594 (14)	0.34937 (12)	0.73347 (12)	0.0133 (3)	
C5	0.60417 (15)	0.35855 (12)	0.72784 (13)	0.0153 (3)	
C6	0.56565 (17)	0.17576 (14)	0.58648 (15)	0.0209 (3)	
C7	0.77354 (16)	0.61333 (15)	0.91932 (15)	0.0213 (3)	
C8	0.26914 (14)	0.32159 (12)	0.59847 (12)	0.0135 (3)	
C9	0.16677 (15)	0.19821 (12)	0.52680 (13)	0.0147 (3)	
C10	0.07261 (15)	0.17160 (12)	0.40490 (13)	0.0153 (3)	
C11	0.07936 (15)	0.26965 (13)	0.35266 (12)	0.0142 (3)	
C12	0.18128 (15)	0.39416 (13)	0.42297 (13)	0.0155 (3)	
C13	0.27411 (15)	0.41876 (13)	0.54497 (13)	0.0155 (3)	
C14	0.62129 (15)	0.92206 (13)	1.09772 (12)	0.0144 (3)	
C15	0.40117 (15)	0.90446 (13)	1.12011 (12)	0.0149 (3)	
C16	0.46382 (15)	1.02900 (13)	1.18448 (12)	0.0149 (3)	
C17	0.62246 (15)	1.11457 (12)	1.21918 (12)	0.0142 (3)	
C18	0.37813 (15)	1.08432 (13)	1.22405 (13)	0.0179 (3)	
C19	0.37283 (19)	1.26713 (15)	1.32473 (17)	0.0253 (3)	
C20	0.24606 (16)	0.81093 (14)	1.07923 (14)	0.0209 (3)	
C21	0.72322 (14)	1.15937 (12)	1.35558 (12)	0.0134 (3)	
C22	0.81179 (15)	1.28805 (12)	1.41893 (13)	0.0154 (3)	
C23	0.90581 (15)	1.32811 (12)	1.54268 (13)	0.0160 (3)	
C24	0.91207 (14)	1.23915 (13)	1.60557 (12)	0.0146 (3)	
C25	0.82479 (15)	1.10980 (13)	1.54369 (13)	0.0146 (3)	
C26	0.73188 (14)	1.07151 (12)	1.41985 (13)	0.0140 (3)	
N1	0.53814 (13)	0.59703 (11)	0.90574 (11)	0.0163 (3)	
N2	0.31652 (13)	0.42046 (11)	0.80401 (11)	0.0153 (3)	
N3	0.48323 (13)	0.85228 (11)	1.08401 (11)	0.0162 (3)	
N4	0.68018 (13)	1.04716 (11)	1.15386 (11)	0.0154 (3)	
O1	0.33372 (11)	0.59969 (9)	0.91804 (9)	0.0185 (2)	
O2	0.73271 (11)	0.40171 (9)	0.74845 (10)	0.0225 (2)	
O3	0.50735 (11)	0.24088 (9)	0.64955 (9)	0.0191 (2)	
O4	−0.01614 (11)	0.23932 (9)	0.23188 (9)	0.0178 (2)	
O5	−0.03765 (13)	0.44197 (10)	0.16905 (11)	0.0253 (3)	
O6	0.68445 (10)	0.86842 (9)	1.05711 (9)	0.0181 (2)	
O7	0.25613 (12)	1.02650 (11)	1.21377 (13)	0.0372 (3)	
O8	0.45133 (11)	1.21067 (9)	1.27839 (10)	0.0215 (2)	
O9	1.00441 (11)	1.28271 (9)	1.72845 (9)	0.0182 (2)	
O10	1.02933 (13)	1.10134 (11)	1.82525 (11)	0.0279 (3)	
H2	0.3460 (16)	0.2653 (14)	0.7513 (13)	0.012 (4)*	
H4	0.6248 (16)	1.1903 (14)	1.1908 (14)	0.013 (4)*	
H6C	0.801 (2)	0.695 (2)	0.9685 (19)	0.042 (6)*	
H6A	0.6325 (19)	0.1504 (15)	0.6451 (15)	0.020 (4)*	
H6B	0.618 (2)	0.2300 (17)	0.5497 (17)	0.032 (5)*	
H7A	0.473 (2)	0.1020 (18)	0.5170 (18)	0.037 (5)*	
H7B	0.810 (2)	0.6203 (19)	0.8581 (19)	0.043 (6)*	
H7C	0.823 (2)	0.572 (2)	0.971 (2)	0.050 (6)*	
H9	0.1627 (17)	0.1297 (15)	0.5647 (14)	0.014 (4)*	
H10	0.0015 (18)	0.0858 (15)	0.3535 (15)	0.017 (4)*	
H12	0.1873 (18)	0.4638 (16)	0.3874 (15)	0.023 (4)*	
H13	0.3430 (18)	0.5067 (16)	0.5956 (15)	0.022 (4)*	
H19A	0.272 (2)	1.2425 (19)	1.2587 (19)	0.041 (5)*	
H19B	0.430 (2)	1.355 (2)	1.354 (2)	0.050 (6)*	
H19C	0.369 (2)	1.2410 (18)	1.3956 (18)	0.035 (5)*	
H20A	0.224 (2)	0.8036 (18)	1.1506 (19)	0.040 (5)*	
H20B	0.176 (2)	0.8369 (18)	1.0264 (18)	0.035 (5)*	
H20C	0.232 (2)	0.725 (2)	1.0365 (19)	0.043 (6)*	
H22	0.8056 (18)	1.3477 (16)	1.3726 (15)	0.022 (4)*	
H23	0.9677 (18)	1.4181 (16)	1.5872 (15)	0.019 (4)*	
H25	0.8305 (18)	1.0473 (15)	1.5905 (15)	0.020 (4)*	
H26	0.6728 (17)	0.9814 (15)	1.3765 (14)	0.014 (4)*	
H1N	0.587 (2)	0.6755 (18)	0.9547 (16)	0.025 (4)*	
H3N	0.442 (2)	0.7732 (19)	1.0363 (18)	0.034 (5)*	
H2N	0.220 (2)	0.3872 (16)	0.7813 (16)	0.024 (4)*	
H4N	0.769 (2)	1.0913 (17)	1.1660 (16)	0.025 (5)*	
H1O	−0.017 (2)	0.308 (2)	0.2135 (19)	0.045 (6)*	
H2O	1.007 (2)	1.218 (2)	1.759 (2)	0.047 (6)*	
H3O	−0.126 (3)	0.447 (2)	0.147 (2)	0.049 (6)*	
H4O	0.033 (2)	0.513 (2)	0.1929 (19)	0.040 (6)*	
H5O	0.954 (3)	1.045 (2)	1.824 (2)	0.050 (6)*	
H6O	1.115 (3)	1.097 (2)	1.870 (2)	0.048 (6)*	

### Atomic displacement parameters (Å^2^)

**Table:**

	*U*^11^	*U*^22^	*U*^33^	*U*^12^	*U*^13^	*U*^23^
C1	0.0135 (7)	0.0148 (6)	0.0126 (6)	0.0056 (5)	0.0053 (5)	0.0037 (5)
C2	0.0130 (7)	0.0171 (7)	0.0149 (7)	0.0068 (5)	0.0060 (5)	0.0064 (5)
C3	0.0112 (6)	0.0138 (6)	0.0153 (7)	0.0050 (5)	0.0053 (5)	0.0056 (5)
C4	0.0118 (6)	0.0104 (6)	0.0175 (7)	0.0041 (5)	0.0075 (5)	0.0021 (5)
C5	0.0140 (7)	0.0136 (6)	0.0176 (7)	0.0055 (5)	0.0065 (6)	0.0058 (5)
C6	0.0216 (8)	0.0187 (7)	0.0271 (8)	0.0102 (6)	0.0155 (7)	0.0031 (6)
C7	0.0136 (7)	0.0198 (7)	0.0219 (8)	0.0025 (6)	0.0057 (6)	−0.0002 (6)
C8	0.0104 (6)	0.0133 (6)	0.0171 (7)	0.0055 (5)	0.0071 (5)	0.0021 (5)
C9	0.0139 (7)	0.0123 (6)	0.0196 (7)	0.0061 (5)	0.0091 (6)	0.0039 (5)
C10	0.0120 (7)	0.0112 (6)	0.0192 (7)	0.0035 (5)	0.0067 (6)	−0.0008 (5)
C11	0.0111 (6)	0.0171 (7)	0.0147 (7)	0.0067 (5)	0.0066 (5)	0.0019 (5)
C12	0.0140 (7)	0.0137 (6)	0.0190 (7)	0.0056 (5)	0.0079 (6)	0.0051 (5)
C13	0.0112 (6)	0.0111 (6)	0.0201 (7)	0.0026 (5)	0.0061 (6)	0.0010 (5)
C14	0.0125 (6)	0.0160 (6)	0.0116 (6)	0.0059 (5)	0.0026 (5)	0.0034 (5)
C15	0.0121 (7)	0.0191 (7)	0.0117 (6)	0.0066 (5)	0.0038 (5)	0.0041 (5)
C16	0.0119 (7)	0.0173 (7)	0.0157 (7)	0.0063 (5)	0.0060 (5)	0.0052 (5)
C17	0.0137 (7)	0.0128 (6)	0.0169 (7)	0.0061 (5)	0.0074 (6)	0.0038 (5)
C18	0.0142 (7)	0.0180 (7)	0.0205 (7)	0.0067 (6)	0.0072 (6)	0.0050 (6)
C19	0.0263 (9)	0.0200 (8)	0.0398 (10)	0.0132 (7)	0.0224 (8)	0.0086 (7)
C20	0.0140 (7)	0.0191 (7)	0.0216 (8)	0.0023 (6)	0.0065 (6)	−0.0007 (6)
C21	0.0100 (6)	0.0131 (6)	0.0173 (7)	0.0048 (5)	0.0074 (5)	0.0015 (5)
C22	0.0150 (7)	0.0126 (6)	0.0207 (7)	0.0057 (5)	0.0104 (6)	0.0041 (5)
C23	0.0134 (7)	0.0098 (6)	0.0214 (7)	0.0021 (5)	0.0089 (6)	−0.0012 (5)
C24	0.0101 (6)	0.0163 (6)	0.0153 (7)	0.0050 (5)	0.0062 (5)	−0.0008 (5)
C25	0.0120 (6)	0.0142 (6)	0.0187 (7)	0.0058 (5)	0.0079 (6)	0.0041 (5)
C26	0.0110 (6)	0.0095 (6)	0.0190 (7)	0.0028 (5)	0.0068 (5)	0.0008 (5)
N1	0.0130 (6)	0.0115 (6)	0.0173 (6)	0.0017 (5)	0.0051 (5)	−0.0016 (5)
N2	0.0101 (6)	0.0147 (6)	0.0172 (6)	0.0030 (5)	0.0060 (5)	0.0001 (5)
N3	0.0128 (6)	0.0126 (6)	0.0178 (6)	0.0032 (5)	0.0053 (5)	−0.0005 (5)
N4	0.0119 (6)	0.0142 (6)	0.0175 (6)	0.0034 (5)	0.0071 (5)	0.0013 (5)
O1	0.0154 (5)	0.0168 (5)	0.0201 (5)	0.0061 (4)	0.0074 (4)	−0.0002 (4)
O2	0.0129 (5)	0.0211 (5)	0.0301 (6)	0.0053 (4)	0.0101 (4)	0.0021 (4)
O3	0.0158 (5)	0.0139 (5)	0.0264 (6)	0.0052 (4)	0.0114 (4)	0.0000 (4)
O4	0.0158 (5)	0.0165 (5)	0.0148 (5)	0.0042 (4)	0.0039 (4)	0.0023 (4)
O5	0.0141 (6)	0.0149 (5)	0.0356 (6)	0.0039 (5)	0.0036 (5)	0.0026 (5)
O6	0.0150 (5)	0.0169 (5)	0.0202 (5)	0.0072 (4)	0.0073 (4)	0.0008 (4)
O7	0.0200 (6)	0.0253 (6)	0.0613 (9)	0.0033 (5)	0.0247 (6)	−0.0035 (6)
O8	0.0200 (5)	0.0154 (5)	0.0342 (6)	0.0086 (4)	0.0173 (5)	0.0051 (4)
O9	0.0152 (5)	0.0160 (5)	0.0152 (5)	0.0027 (4)	0.0041 (4)	−0.0005 (4)
O10	0.0135 (6)	0.0255 (6)	0.0367 (7)	0.0048 (5)	0.0054 (5)	0.0119 (5)

### Geometric parameters (Å, º)

**Table:**

C1—O1	1.2481 (17)	C15—C20	1.4984 (19)
C1—N2	1.3342 (17)	C16—C18	1.4659 (19)
C1—N1	1.3653 (18)	C16—C17	1.5177 (19)
C2—C3	1.3579 (19)	C17—N4	1.4776 (17)
C2—N1	1.3872 (18)	C17—C21	1.5188 (19)
C2—C7	1.4973 (19)	C17—H4	0.994 (15)
C3—C5	1.4647 (19)	C18—O7	1.2105 (18)
C3—C4	1.5218 (18)	C18—O8	1.3444 (17)
C4—N2	1.4745 (17)	C19—O8	1.4499 (17)
C4—C8	1.5182 (19)	C19—H19A	0.99 (2)
C4—H2	1.009 (15)	C19—H19B	0.94 (2)
C5—O2	1.2127 (17)	C19—H19C	0.98 (2)
C5—O3	1.3548 (17)	C20—H20A	1.01 (2)
C6—O3	1.4491 (17)	C20—H20B	0.96 (2)
C6—H6A	0.968 (17)	C20—H20C	1.00 (2)
C6—H6B	0.989 (19)	C21—C26	1.3931 (19)
C6—H7A	1.03 (2)	C21—C22	1.3946 (19)
C7—H6C	0.95 (2)	C22—C23	1.384 (2)
C7—H7B	0.98 (2)	C22—H22	0.968 (17)
C7—H7C	0.96 (2)	C23—C24	1.391 (2)
C8—C9	1.3917 (19)	C23—H23	0.975 (17)
C8—C13	1.3918 (19)	C24—O9	1.3699 (17)
C9—C10	1.384 (2)	C24—C25	1.3965 (19)
C9—H9	0.982 (16)	C25—C26	1.383 (2)
C10—C11	1.3904 (19)	C25—H25	0.999 (17)
C10—H10	0.972 (16)	C26—H26	0.971 (16)
C11—O4	1.3696 (16)	N1—H1N	0.878 (19)
C11—C12	1.3966 (19)	N2—H2N	0.890 (19)
C12—C13	1.385 (2)	N3—H3N	0.88 (2)
C12—H12	0.976 (17)	N4—H4N	0.867 (19)
C13—H13	0.983 (17)	O4—H1O	0.86 (2)
C14—O6	1.2472 (17)	O5—H3O	0.91 (2)
C14—N4	1.3386 (18)	O5—H4O	0.83 (2)
C14—N3	1.3706 (18)	O9—H2O	0.90 (2)
C15—C16	1.3523 (19)	O10—H5O	0.86 (3)
C15—N3	1.3869 (18)	O10—H6O	0.90 (2)
			
O1—C1—N2	122.43 (12)	N4—C17—C16	109.46 (11)
O1—C1—N1	120.57 (12)	N4—C17—C21	109.83 (11)
N2—C1—N1	116.97 (12)	C16—C17—C21	113.17 (11)
C3—C2—N1	119.80 (12)	N4—C17—H4	106.2 (9)
C3—C2—C7	126.65 (13)	C16—C17—H4	109.2 (9)
N1—C2—C7	113.53 (12)	C21—C17—H4	108.7 (9)
C2—C3—C5	120.57 (12)	O7—C18—O8	121.30 (13)
C2—C3—C4	121.63 (12)	O7—C18—C16	126.02 (13)
C5—C3—C4	117.78 (11)	O8—C18—C16	112.66 (12)
N2—C4—C8	108.71 (10)	O8—C19—H19A	109.5 (12)
N2—C4—C3	109.65 (11)	O8—C19—H19B	106.2 (14)
C8—C4—C3	115.47 (11)	H19A—C19—H19B	112.9 (18)
N2—C4—H2	105.7 (9)	O8—C19—H19C	110.4 (11)
C8—C4—H2	107.5 (9)	H19A—C19—H19C	111.3 (16)
C3—C4—H2	109.4 (9)	H19B—C19—H19C	106.4 (17)
O2—C5—O3	121.53 (13)	C15—C20—H20A	111.6 (12)
O2—C5—C3	127.41 (13)	C15—C20—H20B	111.1 (12)
O3—C5—C3	111.05 (11)	H20A—C20—H20B	106.6 (16)
O3—C6—H6A	107.7 (10)	C15—C20—H20C	109.4 (12)
O3—C6—H6B	112.2 (11)	H20A—C20—H20C	107.8 (16)
H6A—C6—H6B	110.0 (14)	H20B—C20—H20C	110.3 (16)
O3—C6—H7A	103.6 (11)	C26—C21—C22	118.39 (13)
H6A—C6—H7A	115.1 (14)	C26—C21—C17	120.28 (12)
H6B—C6—H7A	108.1 (15)	C22—C21—C17	121.31 (12)
C2—C7—H6C	110.7 (12)	C23—C22—C21	121.08 (13)
C2—C7—H7B	112.1 (12)	C23—C22—H22	121.6 (10)
H6C—C7—H7B	109.6 (17)	C21—C22—H22	117.3 (10)
C2—C7—H7C	109.8 (13)	C22—C23—C24	119.74 (12)
H6C—C7—H7C	107.8 (18)	C22—C23—H23	121.3 (9)
H7B—C7—H7C	106.7 (17)	C24—C23—H23	119.0 (10)
C9—C8—C13	118.41 (13)	O9—C24—C23	118.02 (12)
C9—C8—C4	120.22 (12)	O9—C24—C25	121.92 (12)
C13—C8—C4	121.27 (12)	C23—C24—C25	120.06 (13)
C10—C9—C8	121.23 (13)	C26—C25—C24	119.37 (12)
C10—C9—H9	120.3 (9)	C26—C25—H25	121.7 (10)
C8—C9—H9	118.5 (9)	C24—C25—H25	118.9 (10)
C9—C10—C11	119.66 (12)	C25—C26—C21	121.36 (12)
C9—C10—H10	122.1 (9)	C25—C26—H26	119.4 (9)
C11—C10—H10	118.2 (9)	C21—C26—H26	119.3 (9)
O4—C11—C10	117.81 (12)	C1—N1—C2	123.79 (12)
O4—C11—C12	122.17 (12)	C1—N1—H1N	115.9 (12)
C10—C11—C12	120.02 (13)	C2—N1—H1N	120.3 (12)
C13—C12—C11	119.37 (13)	C1—N2—C4	126.69 (12)
C13—C12—H12	119.8 (10)	C1—N2—H2N	115.7 (11)
C11—C12—H12	120.8 (10)	C4—N2—H2N	115.6 (11)
C12—C13—C8	121.30 (12)	C14—N3—C15	123.93 (12)
C12—C13—H13	119.6 (10)	C14—N3—H3N	114.0 (13)
C8—C13—H13	119.1 (10)	C15—N3—H3N	120.9 (13)
O6—C14—N4	123.48 (13)	C14—N4—C17	126.51 (12)
O6—C14—N3	119.81 (12)	C14—N4—H4N	117.2 (12)
N4—C14—N3	116.70 (12)	C17—N4—H4N	115.1 (12)
C16—C15—N3	119.54 (12)	C5—O3—C6	115.32 (11)
C16—C15—C20	127.09 (13)	C11—O4—H1O	109.9 (14)
N3—C15—C20	113.36 (12)	H3O—O5—H4O	112.6 (19)
C15—C16—C18	119.77 (12)	C18—O8—C19	113.98 (11)
C15—C16—C17	122.02 (12)	C24—O9—H2O	111.0 (15)
C18—C16—C17	118.21 (12)	H5O—O10—H6O	113 (2)
			
N1—C2—C3—C5	178.24 (12)	C15—C16—C18—O8	−175.05 (12)
C7—C2—C3—C5	−3.6 (2)	C17—C16—C18—O8	5.71 (18)
N1—C2—C3—C4	−2.98 (19)	N4—C17—C21—C26	−68.68 (15)
C7—C2—C3—C4	175.15 (13)	C16—C17—C21—C26	53.97 (16)
C2—C3—C4—N2	−5.41 (17)	N4—C17—C21—C22	109.60 (14)
C5—C3—C4—N2	173.40 (11)	C16—C17—C21—C22	−127.75 (13)
C2—C3—C4—C8	117.77 (14)	C26—C21—C22—C23	−0.2 (2)
C5—C3—C4—C8	−63.42 (15)	C17—C21—C22—C23	−178.55 (12)
C2—C3—C5—O2	−3.9 (2)	C21—C22—C23—C24	−0.3 (2)
C4—C3—C5—O2	177.25 (13)	C22—C23—C24—O9	−178.78 (12)
C2—C3—C5—O3	177.24 (12)	C22—C23—C24—C25	0.6 (2)
C4—C3—C5—O3	−1.58 (17)	O9—C24—C25—C26	179.01 (12)
N2—C4—C8—C9	−108.73 (13)	C23—C24—C25—C26	−0.4 (2)
C3—C4—C8—C9	127.59 (13)	C24—C25—C26—C21	−0.2 (2)
N2—C4—C8—C13	67.52 (16)	C22—C21—C26—C25	0.5 (2)
C3—C4—C8—C13	−56.15 (17)	C17—C21—C26—C25	178.83 (12)
C13—C8—C9—C10	0.6 (2)	O1—C1—N1—C2	−179.62 (12)
C4—C8—C9—C10	176.94 (12)	N2—C1—N1—C2	2.45 (19)
C8—C9—C10—C11	−0.3 (2)	C3—C2—N1—C1	5.2 (2)
C9—C10—C11—O4	−179.75 (12)	C7—C2—N1—C1	−173.19 (13)
C9—C10—C11—C12	0.3 (2)	O1—C1—N2—C4	169.09 (12)
O4—C11—C12—C13	179.50 (12)	N1—C1—N2—C4	−13.0 (2)
C10—C11—C12—C13	−0.6 (2)	C8—C4—N2—C1	−113.04 (14)
C11—C12—C13—C8	0.8 (2)	C3—C4—N2—C1	14.04 (18)
C9—C8—C13—C12	−0.8 (2)	O6—C14—N3—C15	−176.36 (12)
C4—C8—C13—C12	−177.17 (12)	N4—C14—N3—C15	2.68 (19)
N3—C15—C16—C18	−179.33 (12)	C16—C15—N3—C14	−7.4 (2)
C20—C15—C16—C18	1.5 (2)	C20—C15—N3—C14	171.83 (12)
N3—C15—C16—C17	−0.1 (2)	O6—C14—N4—C17	−170.82 (12)
C20—C15—C16—C17	−179.25 (13)	N3—C14—N4—C17	10.18 (19)
C15—C16—C17—N4	10.20 (18)	C16—C17—N4—C14	−15.85 (18)
C18—C16—C17—N4	−170.57 (11)	C21—C17—N4—C14	108.97 (14)
C15—C16—C17—C21	−112.65 (14)	O2—C5—O3—C6	−4.45 (19)
C18—C16—C17—C21	66.57 (15)	C3—C5—O3—C6	174.46 (11)
C15—C16—C18—O7	6.7 (2)	O7—C18—O8—C19	1.7 (2)
C17—C16—C18—O7	−172.58 (15)	C16—C18—O8—C19	−176.70 (12)

### Hydrogen-bond geometry (Å, º)

Cg1 and Cg2 are the centroids of the C8–C13 and C21–C26 rings, respectively.

**Table:**

*D*—H···*A*	*D*—H	H···*A*	*D*···*A*	*D*—H···*A*
N1—H1*N*···O6	0.878 (19)	2.10 (2)	2.9762 (16)	173.1 (17)
N3—H3*N*···O1	0.88 (2)	1.98 (2)	2.8626 (16)	174 (2)
N2—H2*N*···O9^i^	0.89 (2)	2.02 (2)	2.8971 (19)	170.0 (18)
N4—H4*N*···O4^ii^	0.87 (2)	2.13 (2)	2.9738 (19)	163.4 (18)
O4—H1*O*···O5	0.87 (2)	1.78 (2)	2.6473 (16)	175 (2)
O9—H2*O*···O10	0.90 (2)	1.73 (2)	2.6189 (17)	174.7 (19)
O5—H4*O*···O2^iii^	0.84 (2)	2.15 (2)	2.8549 (19)	141 (2)
O5—H3*O*···O1^iv^	0.91 (3)	1.91 (3)	2.786 (2)	162 (2)
C20—H20*C*···O5^iv^	1.00 (2)	2.56 (2)	3.332 (2)	133.7 (17)
C26—H26···O3^v^	0.971 (17)	2.571 (18)	3.4607 (18)	152.4 (14)
C6—H6*B*···*Cg*2^vi^	0.989 (19)	2.70 (2)	3.392 (2)	127.1 (16)
C19—H19*C*···*Cg*1^vii^	0.98 (2)	2.84 (2)	3.395 (2)	116.5 (16)

Symmetry codes: (i) *x*−1, *y*−1, *z*−1; (ii) *x*+1, *y*+1, *z*+1; (iii) −*x*+1, −*y*+1, −*z*+1; (iv) −*x*, −*y*+1, −*z*+1; (v) −*x*+1, −*y*+1, −*z*+2; (vi) *x*, *y*, *z*−1; (vii) *x*, *y*+1, *z*+1.
